# Traumatic Brain Injury Related Hospitalization and Mortality in California

**DOI:** 10.1155/2013/143092

**Published:** 2013-11-13

**Authors:** Clint Lagbas, Shahrzad Bazargan-Hejazi, Magda Shaheen, Dulcie Kermah, Deyu Pan

**Affiliations:** ^1^Charles R Drew University of Medicine & Science, David Geffen School of Medicine at UCLA, 1731 East 120th Street, Los Angeles, CA 90059, USA; ^2^Department of Psychiatry, College of Medicine, Charles R Drew University of Medicine, 1731 East 120th Street, Los Angeles, CA 90059, USA; ^3^Semel Institute for Neuroscience and Human Behavior, David Geffen School of Medicine at UCLA, USA; ^4^Charles R. Drew University of Medicine and Science, College of Medicine, 1731 East 120th Street, Los Angeles, CA 90059, USA; ^5^Department of Research, Charles R. Drew University of Medicine and Science, 1731 East 120th Street, Los Angeles, CA 90059, USA

## Abstract

*Objective*. The aim of this study is to describe the traumatic brain injury (TBI) population and causes and identify factors associated with TBI hospitalizations and mortality in California. *Methods*. This is a cross-sectional study of 61,188 patients with TBI from the California Hospital Discharge Data 2001 to 2009. We used descriptive, bivariate, and multivariate analyses in SAS version 9.3. *Results*. TBI-related hospitalizations decreased by 14% and mortality increased by 19% from 2001 to 2009. The highest percentages of TBI hospitalizations were due to other causes (38.4%), falls (31.2%), being of age ≥75 years old (37.2%), being a males (58.9%), and being of Medicare patients (44%). TBIs due to falls were found in those age ≤4 years old (53.5%), ≥75 years old (44.0%), and females (37.2%). TBIs due to assaults were more frequent in Blacks (29.0%). TBIs due to motor vehicle accidents were more frequent in 15–19 and 20–24 age groups (48.7% and 48.6%, resp.) and among Hispanics (27.8%). Higher odds of mortality were found among motor vehicle accident category (adjusted odds ratio (AOR): 1.27, 95% CI: 1.14–1.41); males (AOR: 1.36, 95% CI: 1.27–1.46); and the ≥75-year-old group (AOR: 6.4, 95% CI: 4.9–8.4). *Conclusions*. Our findings suggest a decrease in TBI-related hospitalizations but an increase in TBI-related mortality during the study period. The majority of TBI-related hospitalizations was due to other causes and falls and was more frequent in the older, male, and Medicare populations. The higher likelihood of TBI-related mortalities was found among elderly male ≥75 years old who had motor vehicle accidents. Our data can inform practitioners, prevention planners, educators, service sectors, and policy makers who aim to reduce the burden of TBI in the community. Implications for interventions are discussed.

## 1. Introduction

Traumatic brain injury (TBI) is defined by the Centers for Disease Control and Prevention (CDC) as an injury to the head resulting from blunt or penetrating trauma or from acceleration-deceleration of force causing neurological or neuropsychological abnormalities, such as altered level of consciousness, intracranial lesion, memory loss, skull fracture, or death [1]. In the United States, during 2002–2007, approximately 1.7 million Americans sustained some kind of traumatic brain injury, annually. This led to 275,000 hospitalizations (16%), over 1.3,000,000 million visits to the Emergency Department (81%), 52,000 deaths (3.0%), and 124,000 disabilities [2, 3]. 

TBI-related direct and indirect costs, including medical costs and loss of productivity, totaled an estimated $60 billion in the United States, annually [4]. Findings from the Centers for Disease Control and Prevention data suggest that TBI-related hospitalization (TBI-H) and TBI-related mortality (TBI-M) increased by 3.5% and 19.5%, respectively, from 2002 to 2006 [3]. In a study of the 12 states participating in Centers for Disease Control and Prevention TBI-H surveillance in 2002, California and New York accounted for approximately 54% of all cases reported for TBI-H [3]. However, there is a dearth of research at the state level, especially for California which had 75.8/100,000 cases of TBI-H in 2002 [5]. 

Our study aim is to examine sociodemographic variables related to TBI-H, TBI-related causes (TBI-C), and TBI-M in California. Based on the existing literature, we hypothesize that there will be a statistically significant age, gender, and racial differences with respect to TBI-H, TBI-C, and TBI-M. Findings from this study can inform health policy makers and health promotion programmers to identify populations at greater risk of TBI-H and TBI-M in California. In addition, identifying sociodemographic disparities in the experience of TBI can inform practitioners, prevention planners, educators, and service sectors who aim to reduce the burden of TBI in their community.

## 2. Methods

### 2.1. Design and Study Data

**Figure 1:**
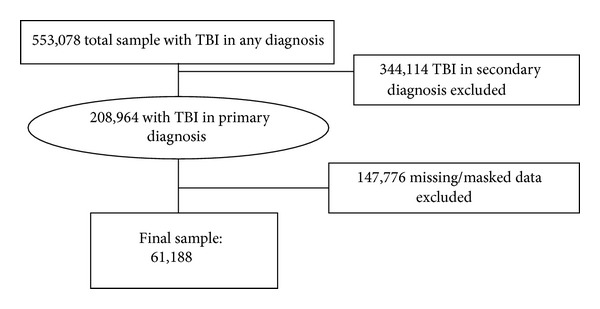
Traumatic brain injury related hospitalization and mortality in california.

This was a cross-sectional study of data obtained from the Office of Statewide Health Planning and Development (OSHPD), a database that contains a summary of all the inpatient hospital discharges in California and is used for billing and payment services. For this study we used California hospital discharge data for 2001–2009 (See Figure 1). The data provided by the OSPHD includes patient demographics (age, sex, and race/ethnicity), diagnostic codes, source of payment (i.e., insurance), admission year, length of hospital stay, and disposition. All patients were eligible to be included in the study. Only patients with complete data in all the variables were included in the analysis (*n* = 61, 188).

We used the International Classification of Diseases, 9th Revision, and Clinical Modification (ICD-9-CM) to identify TBI-H (Table 1). To identify TBI-M, we used ICD-10-CM. To classify TBI-C, we used CDC framework for injury categorization using E codes that are grouped into five categories: motor vehicle accidents; falls (unintentional and undetermined); assaults (including firearms and other methods); struck by and struck against (including homicide and injury purposely inflicted by another person).

Age in years was included as 11 groups (0–4, 5–9, 10–14, 15–19, 20–24, 25–34, 35–44, 45–54, 55–64, 65–74, and ≥75). Gender was included as male and female. Race/ethnicity variable included White, Black, Hispanic, Asian, and others. Insurance variable was categorized as Medicare, Medicaid, private, worker compensation, and others. Length of hospital stay was the number of days from admission to disposition. The severity variable was developed using the aforementioned ICD-9 codes and was categorized as minor, moderate, serious, severe, and critical [6].

### 2.2. Date Analysis

All analyses were obtained using Statistical Analysis Software, SAS version 9.3. We used frequency (count and percentage) to depict the overall characteristics of the sample for the categorical variables (age, sex, race/ethnicity, insurance status, length of hospital stay, and years of admission). We conducted bivariate analysis using the chi square test to determine the statistical difference in the outcome variables (TBI-H and TBI-M) by the main independent variable (TBI-C) and the other independent variables (age, gender, race/ethnicity, length of stay, severity, insurance status, and admission year). We used unadjusted logistic regressions to determine the association between each independent variable and the TBI-M. In addition, we performed multiple logistic regression to test the independent association between study predictor variables and TBI-M while controlling for the other variables in the model (i.e., age, gender, race/ethnicity, admission year, length of stay, insurance status, and severity). Unadjusted and adjusted odds ratios and 95% confidence intervals (CIs) are presented, and statistical significance is considered at *P* value ≤0.05.

## 3. Results

### 3.1. Sample Characteristics

The study included 61,188 hospital admissions. The average age of the population was 54.9 years, and standard deviation was 27.5 years.  Table 2 illustrates that from 2001 to 2009, the percentage of TBI-H decreased from 10.3% to 8.9%, and 7.7% of patients did not survive their injuries. Our findings also show that, during 2001–2009, the leading causes of TBI-H in California were, in descending order, other causes (38.4%), falls (31.5%), and motor vehicle accidents (19.6%). The highest percentages of TBI-H occurred in those of 75 years, and older (37.2%), and nearly half of patients had Medicare (44.0%). Males had more TBI-H than females (58.9% versus 41.1%). Whites had the highest percentage of TBI-H, more than all the other racial groups combined. The average length of hospital stay was 5.9 days, the standard deviation was 9.2 days, and the median was 3 days. More than half of all admissions (57.7%) were for six days or longer. About two thirds of admissions were evaluated as serious to critical (65.1%).

### 3.2. Factors Associated with TBI-Related Hospitalization

Table 3 presents patterns of TBI-H by external causes and demographic variables. There was a significant association between age group, gender, race, insurance and admission year, length of stay, and severity of injury and causes of TBI-H (*P* < 0.0001). Falls were the leading cause of TBI-H in those of 0–4 years of age (53.5%) and among females (37.2%). Motor vehicle accidents were the leading causes of hospitalization in 15–19 and 20–24 age groups (48.7% and 48.6%, resp.) and among Hispanics (27.8%). Assaults were the leading cause of TBI-H in men (15.0%) versus women (3.5%). Among Blacks, assaults were the leading cause of TBI-H (29.0%).

### 3.3. Factors Associated with TBI-Related Mortality

Table 4 presents mortality percentages as well as unadjusted and adjusted odds ratios of TBI-M in California from 2001 to 2009. Mortality increased from 6.9% in 2001 to 8.2% in 2009. Mortality was higher among those who suffered an assault, among those of 75 years age and older, males, Whites, and those who had serious to critical injuries. After adjusting for other independent variables, patients were more likely to die from TBI caused by motor vehicle accidents compared to those who were “struck by” (adjusted odds ratio [AOR] = 1.27, 95% CI = 1.14–1.41; *P* < 0.0001). Also, those of age ≥75 (AOR = 6.4, 95% CI = 4.9–8.4; *P* < 0.0001) and patients with Medicare (AOR = 1.52; 95% CI = 1.32–1.75; *P* < 0.0001) had higher odds of dying from TBI compared to other groups. Males were more likely to die from TBI compared to females (AOR = 1.36, 95% CI = 1.27–1.46, *P* < 0.0001). Blacks suffered higher mortality compared to Whites, but this was not statistically significant (AOR = 1.10, 95% CI = 0.945–1.285, *P* < 0.22).

## 4. Discussion

In this study, we identified trends as well as sociodemographic factors and causes of injuries related to TBI hospitalization and mortality. Our findings show that, from 2001 to 2009, the percentage of TBI-H in California decreased while mortality from such injuries increased. The lower percentage of TBI-H suggests that those with critical injuries may succumb before reaching a hospital, due to the lack of transportation or accessibility to adequate care, for example, living at a great distance away. In Trunkey and Blaisdel's “trimodel distribution of trauma deaths,” they refer to this as prehospital deaths [7]. Alternatively, the difference may simply reflect improved, pre-hospital care in California. Future studies can elucidate underlying mechanisms and remediable causes and treat them by the most effective interventions.

We found an increasing trend in TBI-M in our study. This may reflect the loss of the proverbial “golden hour” of trauma care in California that could increase early hospital deaths. It may also suggest “late deaths” due to trauma-related complications such as sepsis [7]. Faul et al. showed that the percentage of TBI-M in United States increased by 3.5%, during 2002–2006 [3]. However, the observed differences could be due to variations in the age distribution of our study population and causes of TBI. For example, our study consisted of 37.2% age ≥75 years, compared to 22% in Faul et al., and included higher percentages of falls and assaults. 

Our findings suggest a high burden of TBI in California, which can lead to substantial long-term cognitive, emotional, and functional disability [8]. It is estimated that 124,000 persons discharged from TBI-related hospitalization developed TBI-related disability one year after the injury [6]. In a 2005 study by Zaloshnja et al., it was estimated that 3.2 million Americans were living with TBI-related long-term disability. Approximately half of these individuals were of ages of 40–49 years old, and 25% were those ≥70 years of age [9]. Our findings suggest the need for quality assessment of TBI-related care in California that will identify areas for tertiary prevention of trauma deaths.

Our findings also show that, in California, the highest percentages of TBI-H were in those of ≥75 years of age, males, Whites, and those who had Medicare coverage. In regard to TBI-M, it was more likely to occur in those of ≥75 years age, male, and those with Medicaid. These findings are similar to previous national studies of TBI [10–14]. For example in 2003, Rutland-Brown et al. showed that those of 65 years of age and older had the highest rates of TBI-related hospitalizations and mortality (234.1 per 100,000 and 38.4 per 100,000, resp.) [2]. With the steady increase in the population of those 65 and older, it seems there is a need to reduce the burden of unintentional falls and motor vehicle-related accidents in this subpopulation [15, 16]. Specifically, primary care providers and emergency physicians are in the unique position of being able to inform elderly individuals and their caregivers of risks associated with fall injuries and motor vehicle crashes. Moreover, evidence-based preventions, which are cost effective and focus on environmental modifications to reduce the risk of unintentional fall in this population, as well as their susceptibility to injury in motor vehicle crashes, are needed [15, 17].

The higher TBI hospitalizations and mortality among male relative to female had been reported in previous studies [3, 5, 14, 18–20]. For example, from 1980 to 1995, the average rate of TBI-related hospitalizations was 1.8 times higher in male than female. In 1994, male death rates were 3.3 times higher than females [21], Fall-related TBI deaths were about 3 times higher in males than females (3.2 versus 1.3 per 100,000), and motor vehicle-traffic related TBI deaths were about 2 times higher in males than females (9.9 versus 4.3 per 100,000) during 1989–1998 [22]. Although reasons for differences in males and females remain unclear, previous research suggested differences in physiological responses and lifestyle-related behaviors [8, 19, 20]. 

Our data showed that Blacks had higher adjusted odds of mortality relative to Whites, but it was not statistically significant. Different from our findings, others have reported racial differences in the incidence of TBI-related hospitalizations and mortality. For example, TBI data from the United States for 1995–2001 identifies that both Blacks and American Indians/Alaska natives as the groups with the highest TBI-related hospitalizations [23, 24]. Also, TBI-related death rates for 1994 were high among Blacks (25.5 per 100,000) than Whites (19 per 100,000) [1]. Data from 1995 to 2001 also point out Blacks as the highest risk group for TBI-associated death [24, 25]. These findings are similar to findings also reported from the IMPACT study [26]. Others have shown that Asian had higher mortality compared to Whites [20, 27, 28]. Differences could be due to the effect of injury on mortality. In one study, excluding cases of assaults resulted in a significant increase of mortality in Asians compared to Whites, suggesting that assaults may have an effect on mortality [28]. In our study, we found that assaults were the leading cause of TBI in Blacks, a finding that was noted in the IMPACT study [26]. A high percentage of assault-related injuries in Blacks emphasize the need to strengthen community-based primary prevention programs as well as clinical prevention services in the community for this group.

According to this California discharge data, falls were the leading cause of TBI-H in those of ages of 0–4 years old and ≥75 years age group, for males and females, as well as Whites and Asians. Findings of studies that examined TBI-H in children are consistent with ours; that is, falls were the leading causes of hospitalizations in children of 0–4 years old [23, 29, 30]. We did not examine the causes of falls among this age group in our study since it was not in the scope of our aims, but previous findings show that the leading causes of falls in infants are falling from bed, stairs, furniture, and chair [30]. Also, for the older age group, others have found that those in the ≥75 years of age group are more subject to falls [2, 16, 30], which is consistent with our data showing a higher percentage of TBI-H caused by falls in those of age ≥75 years old.

Although this study provides a population based analysis of TBI for California, it presents some limitations. More specifically, missing data limited our ability to generalize the findings to the general population. For example, OSPHD does not include data from the Veterans Affairs Medical Center. Also, to protect the identity of individual patients, OSHPD masks some data elements for some encounters. Masking affects age, sex, race, ethnicity, and zip code to varying degrees. Additionally, our numbers do not include patients who were seen in the emergency department or who did not receive injury related care, therefore leading to possible underestimation of the overall percentage of TBI. Also this study focused on aggregated falls and did not count for possible differences in the presentation of individual types of falls. Finally, as helpful as ICD-9 code data are for identifying burden of injury and its causal factors, its use as a research tool is limited due to its potential for missing data. This could significantly impact research outcomes [31]. In our study, however, we did not have any missing ICD codes for the primary diagnosis in the total sample because we selected the sample based on the identifying of ICD-9 codes. 

## 5. Conclusions

Our findings demonstrate the recent trends of risk factors in TBI in California, showing a decrease in TBI-H over the years. They also depict an increase in the percentage of TBI-M during the same period, suggesting that TBI mortality remains a public health challenge in California. In terms of preventing acute, early, and late TBI-related deaths, our findings suggest the need for early identification of potentially fatal TBI injuries. From a primary prevention perspective, our findings also highlight the need for programs geared specifically toward falls, assaults, and motor vehicle accident-related injuries.

In addition, our results suggest differences in demographic factors associated with TBI hospitalizations and mortality in California. These include younger children, older adults, and females, who were at higher risk for fall-related TBIs; younger adults, elderly, and Hispanics, who were at higher risks for motor vehicle accidents-related TBIs; and males and Blacks who had higher risks for assault-related TBIs. Targeted injury preventative strategies for different subgroups are needed to focus on their specific risk for TBI and their common risky behavioral practices [16, 32–34]. Further research is required to determine factors associated with TBI-related emergency department visits and post-TBI hospitalization outcome in California.

## Supplementary Material

Supplementary Material: STROBE 2007 (v4) Statement—Checklist of items that should be included in reports of cross-sectional studies.

## Figures and Tables

**Table 1:** International Classification of Diseases, 9th Revision, Clinical Modification (ICD-9-CM) for identification of TBI-H, TBI-M, and TBI-.

TBI-H	TBI-M	TBI-C	TBI-C	TBI-C	TBI-C
(ICD-9-CM): 800.0–801.9, 803.0–804.9, 850.0–854.1, 950.1–950.3, 995.55, 959.01	ICD-10-CM**: **S01.0–S01.9, S02.0, S02.1, S02.3, S02.7– S02.9, S04.0, S06.0–S06.9, S07.0, S07.1, S07.8, S07.9, S09.7–S09.9, T01.0, T02.0, T04.0, T06.0, T90.1, T90.2, T90.4, T90.5, T90.8, T90.9	Motor vehicle accidents: E810–E825, E958.5, E968.5, and E988.5	Falls: unintentional & undetermined: E880–E886, E888, and E987	Assault: including firearms & other methods: E960–E969 E922, E955.0–CE955.4, E965.0–E965.4, E970, and E985.0–E985.4	Struck by & struck against: including homicide and injury purposely inflicted by another person: E916, E917

**Table 2:** Estimated numbers and percentages of traumatic brain injury related hospitalization in California categorized by causes, demographics, insurance, years of admission, and mortality, 2001–2009.

Patient characteristics	Number (percentage)
Causes	
Motor vehicle	11996 (19.6)
Fall	19113 (31.2)
Assault	6291 (10.3)
Struck by	300 (0.5)
Other	23488 (38.4)
Age	
0–4	3910 (6.4)
5–9	760 (1.2)
10–14	916 (1.5)
15–19	2986 (4.9)
20–24	3256 (5.3)
25–34	4319 (7.1)
35–44	5296 (8.7)
45–54	6580 (10.8)
55–64	4923 (8.1)
65–74	5488 (9.0)
≥75	22754 (37.2)
Gender	
Female	25159 (41.1)
Male	36029 (58.9)
Race	
Blacks	3407 (5.6)
Hispanics	13231 (21.6)
Asians	3317 (5.4)
Other	1001 (1.6)
Whites	40232 (65.8)
Insurance	
Medicare	26942 (44.0)
Medicaid	9556 (15.6)
Private	13666 (22.3)
Other	10128 (16.6)
Workers comp	896 (1.5)
Admission year	
2001	6296 (10.3)
2002	6740 (11.0)
2003	7135 (11.7)
2004	7306 (11.9)
2005	7617 (12.5)
2006	7589 (12.4)
2007	7952 (13.0)
2008	5088 (8.2)
2009	5442 (8.9)
Length of stay	
1-2	25878 (42.3)
3–5	16814 (27.5)
6 or more	18496 (30.2)
Severity	
Minor	8933 (14.7)
Moderate	12341 (20.2)
Serious	18752 (30.7)
Severe	19649 (32.2)
Critical	1320 (2.2)
Mortality	
Alive	56479 (92.3)
Died	4709 (7.7)

**Table 3:** Estimated numbers and percentages of traumatic brain injury-related hospitalizations categorized by external causes and demographics, insurance, admission year, and procedure day in California, 2001–2009.

	Motor vehicle	Fall	Assault	Struck by	Other	*P* value
Age						
0–4	455 (11.6)	2093 (53.5)	322 (8.2)	82 (2.1)	958 (24.5)	<0.0001
5–9	264 (34.7)	236 (31.1)	6 (0.8)	20 (2.6)	234 (30.8)	
10–14	313 (34.2)	182 (19.9)	48 (5.2)	8 (0.9)	365 (39.9)	
15–19	1454 (48.7)	284 (9.5)	573 (19.2)	7 (0.2)	668 (22.4)	
20–24	1582 (48.6)	269 (8.3)	824 (25.3)	13 (0.4)	568 (17.4)	
25–34	1798 (41.6)	435 (10.1)	1173 (27.2)	31 (0.7)	882 (20.4)	
35–44	1688 (31.9)	794 (15.0)	1285 (24.3)	32 (0.6)	1497 (28.3)	
45–54	1631 (24.8)	1289 (19.6)	1279 (19.4)	24 (0.4)	2357 (35.8)	
55–64	994 (20.2)	1431 (29.1)	474 (9.6)	22 (0.5)	2002 (40.7)	
65–74	719 (13.1)	2081 (37.9)	140 (2.6)	21 (0.4)	2527 (46.1)	
≥75	1098 (4.8)	10019 (44.0)	167 (0.7)	40 (0.2)	11430 (50.2)	
Gender						
Female	4507 (17.9)	9356 (37.2)	872 (3.5)	94 (0.4)	10330 (41.1)	<0.0001
Male	7489 (20.8)	9757 (27.1)	5419 (15.0)	206 (0.6)	13158 (36.5)	
Race						
Blacks	725 (21.3)	672 (19.7)	987 (29.0)	22 (0.7)	1001 (29.4)	<0.0001
Hispanics	3672 (27.8)	3274 (24.7)	2508 (19.0)	117 (0.9)	3660 (27.7)	
Asians	476 (14.4)	1086 (32.7)	134 (4.0)	21 (0.6)	1600 (48.2)	
Other	302 (30.2)	265 (26.5)	114 (11.4)	4 (0.4)	316 (31.6)	
Whites	6821 (17.0)	13816 (34.3)	2548 (6.3)	136 (0.3)	16911 (42.0)	
Insurance						
Medicare	1582 (5.9)	11469 (42.6)	482 (1.8)	56 (0.2)	13353 (49.6)	<0.0001
Medicaid	2703 (28.3)	2374 (24.8)	1697 (17.8)	71 (0.7)	2711 (28.4)	
Private	4325 (31.7)	3498 (25.6)	1057 (7.7)	81 (0.6)	4705 (34.4)	
Other	3193 (31.5)	1403 (13.9)	3007 (29.7)	42 (0.4)	2483 (24.5)	
Workers comp	193 (21.5)	369 (41.2)	48 (5.4)	50 (5.6)	236 (26.3)	
Length of stay						
1-2	5584 (46.6)	8200 (42.9)	3320 (52.8)	159 (53)	8615 (36.7)	<0.001
3–5	2595 (21.6)	5679 (29.7)	1402 (22.3)	68 (22.7)	7070 (30.1)	
6 or more	3817 (31.8)	5234 (27.4)	1569 (24.9)	73 (24.3)	7803 (33.2)	
Severity						
Minor	2162 (18.0)	2612 (13.7)	1159 (18.9)	51 (17.0)	2949 (12.6)	<0.0001
Moderate	3746 (31.2)	3641 (19.1)	1279 (20.8)	96 (32.0)	3579 (15.3)	
Serious	4108 (34.2)	5869 (30.7)	2209 (36.0)	104 (34.7)	6462 (27.6)	
Severe	1749 (14.6)	6701 (35.1)	1296 (21.1)	47 (15.7)	9856 (42.0)	
Critical	231 (1.9)	287 (1.5)	201 (3.3)	2 (0.7)	599 (2.5)	
Admission year						
2001	1398 (22.2)	2859 (45.4)	662 (10.5)	42 (0.7)	1335 (21.2)	<0.0001
2002	1484 (22.0)	1977 (29.3)	720 (10.7)	32 (0.5)	2527 (37.5)	
2003	1571 (22.0)	2158 (30.3)	682 (9.6)	38 (0.5)	2686 (38.7)	
2004	1575 (21.6)	2133 (29.2)	725 (9.9)	29 (0.4)	2844 (38.9)	
2005	1418 (18.6)	2137 (28.1)	865 (11.4)	40 (0.5)	3157 (41.5)	
2006	1460 (19.2)	2275 (30.0)	795 (10.5)	47 (0.6)	3012 (39.7)	
2007	1334 (16.8)	2433 (30.6)	774 (9.7)	26 (0.3)	3385 (42.6)	
2008	884 (17.4)	1494 (29.4)	499 (9.8)	27 (0.5)	2184 (42.9)	
2009	869 (16.0)	1640 (30.1)	568 (10.4)	19 (0.4)	2346 (43.1)	

**Table 4:** Estimated number, percentages, unadjusted and adjusted odds ratio, and 95% confidence interval of traumatic brain injury related mortality in California, 2001–2009.

	Mortality		Unadjusted		Adjusted	
	Died	Alive	Odds ratio	95% CI	Odds ratio	95% CI
Causes						
Motor v. accident	726 (6.1)	11270 (93.9)	0.612	0.561–0.667	1.270	1.140–1.415
Fall	1342 (7.0)	17771 (93.0)	0.717	0.669–0.770	0.775	0.716–0.839
Assault	397 (6.3)	5894 (93.7)	0.640	0.573–0.715	0.816	0.706–0.943
Other	2237 (9.5)	21251 (90.5)	0.227	0.107–0.481	0.510	0.232–1.121
Struck by	7 (2.3)	293 (97.7)	Reference	Reference	Reference	Reference
Age						
0–4	93 (2.4)	3817 (97.6)	0.199	0.162–0.246	0.156	0.119–0.204
5–9	16 (2.1)	744 (97.9)	0.176	0.107–0.289	0.147	0.086–0.253
10–14	19 (2.1)	897 (97.9)	0.173	0.110–0.273	0.121	0.073–0.198
15–19	198 (6.6)	2788 (93.4)	0.581	0.500–0.675	0.415	0.336–0.512
20–24	201 (6.2)	3055 (93.8)	0.538	0.464–0.624	0.420	0.340–0.518
25–34	198 (4.6)	4121 (95.4)	0.393	0.339–0.456	0.333	0.271–0.409
35–44	260 (4.9)	5036 (95.1)	0.422	0.370–0.482	0.398	0.331–0.478
45–54	413 (6.3)	6167 (93.7)	0.548	0.492–0.610	0.461	0.395–0.540
55–64	385 (7.8)	4538 (92.2)	0.694	0.620–0.776	0.625	0.538–0.726
65–74	447 (8.2)	5041 (91.8)	0.725	0.653–0.806	0.627	0.558–0.704
≥75	2479 (10.9)	20275 (89.1)	Reference	Reference	Reference	Reference
Gender						
Female	1772 (7.0)	23387 (93.0)	Reference	Reference	Reference	Reference
Male	2937 (8.2)	33092 (91.8)	1.171	1.102–1.245	1.36	1.27–1.46
Race						
Blacks	253 (7.4)	3154 (92.6)	0.879	0.769–1.003	1.102	0.945–1.285
Hispanics	777 (5.9)	12454 (94.1)	0.683	0.630–0.741	0.891	0.805–0.985
Asians	269 (8.1)	3048 (91.9)	0.967	0.849–1.100	0.793	0.689–0.912
Other	44 (4.4)	957 (95.6)	0.504	0.371–0.683	0.723	0.516–1.015
Whites	3366 (8.4)	36866 (91.6)	Reference	Reference	Reference	Reference
Insurance						
Medicare	2749 (10.2)	24193 (89.8)	Reference	Reference	Reference	Reference
Medicaid	723 (7.6)	8833 (92.4)	0.720	0.661–0.784	1.518	1.318–1.748
Private	703 (5.1)	12963 (94.9)	0.477	0.438–0.520	0.767	0.677–0.869
Other	488 (4.8)	9640 (95.2)	0.446	0.403–0.492	0.827	0.707–0.966
Workers comp	46 (5.1)	850 (94.9)	0.476	0.353–0.642	0.848	0.604–1.192
Severity						
Minor	40 (0.4)	8893 (99.6)	Reference	Reference	Reference	Reference
Moderate	117 (0.9)	12224 (99.1)	2.128	1.485–3.050	2.561	1.785–3.67
Serious	1639 (8.7)	17113 (91.3)	21.29	15.54–29.17	30.652	22.34–42.06
Severe	1991 (10.1)	17658 (89.9)	25.07	18.31– 34.32	31.456	22.9–43.18
Critical	902 (68.3)	418 (31.7)	479.75	344.37–668.34	655.24	468.13–917.15
Length of stay						
1-2	2338 (49.7)	23540 (41.7)	Reference	Reference	Reference	Reference
3–5	898 (19.1)	15916 (28.2)	0.568	0.525–0.615	0.320	0.293–0.349
6 or more	1473 (31.3)	17023 (30.1)	0.871	0.814–0.933	0.379	0.351–0.410
Admission yr.						
2001	435 (6.9)	5861 (93.1)	Reference	Reference	Reference	Reference
2002	479 (7.1)	6261 (92.9)	1.031	0.901–1.179	0.969	0.832–1.129
2003	515 (7.2)	6620 (92.8)	1.048	0.918–1.197	1.014	0.873–1.177
2004	554 (7.6)	6752 (92.4)	1.106	0.970–1.259	1.028	0.887–1.191
2005	594 (7.8)	7023 (92.2)	1.140	1.002–1.296	0.940	0.813–1.087
2006	575 (7.6)	7014 (92.4)	1.105	0.971–1.257	0.970	0.839–1.121
2007	638 (8.0)	7337 (92.0)	1.172	1.032–1.330	0.949	0.823–1.094
2008	475 (9.3)	4613 (90.7)	1.387	1.211–1.589	1.209	1.038–1.408
2009	444 (8.2)	4998 (91.8)	1.197	1.043–1.373	1.010	0.866–1.178

## Abbreviations

TBI: Traumatic brain injury
TBI-H: Traumatic brain injury related hospitalization
TBI-C: Traumatic brain injury related causes
TBI-M: Traumatic brain injury related mortality
AOR: Adjusted odds ratio
OSHPD: Office of Statewide Health Planning and Development
ICD-9-CM: International Classification of Diseases, 9th Revision, Clinical Modification
ICD-1-CM: International Classification of Diseases, 10th Revision, Clinical Modification.
